# Newly discovered roles of triosephosphate isomerase including functions within the nucleus

**DOI:** 10.1186/s10020-023-00612-x

**Published:** 2023-01-31

**Authors:** Tracey D. Myers, Michael J. Palladino

**Affiliations:** 1 Department of Pharmacology & Chemical Biology, University of Pittsburgh School of Medicine, Pittsburgh, PA 15261 USA; 2 Pittsburgh Institute for Neurodegenerative Diseases, University of Pittsburgh School of Medicine, Pittsburgh, PA 15261 USA; 3 Center for Neuroscience, University of Pittsburgh School of Medicine, Pittsburgh, PA 15213 USA

**Keywords:** Triosephosphate isomerase (TPI), TPI deficiency, Genetics, Glycolysis, Metabolism, Moonlighting, Gene sharing

## Abstract

Triosephosphate isomerase (TPI) is best known as a glycolytic enzyme that interconverts the 3-carbon sugars dihydroxyacetone phosphate (DHAP) and glyceraldehyde-3-phosphate (G3P). TPI is an essential enzyme that is required for the catabolism of DHAP and a net yield of ATP from anaerobic glucose metabolism. Loss of TPI function results in the recessive disease TPI Deficiency (TPI Df). Recently, numerous lines of evidence suggest the TPI protein has other functions beyond glycolysis, a phenomenon known as moonlighting or gene sharing. Here we review the numerous functions ascribed to TPI, including recent findings of a nuclear role of TPI implicated in cancer pathogenesis and chemotherapy resistance.

## Background

**Fig. 1:**
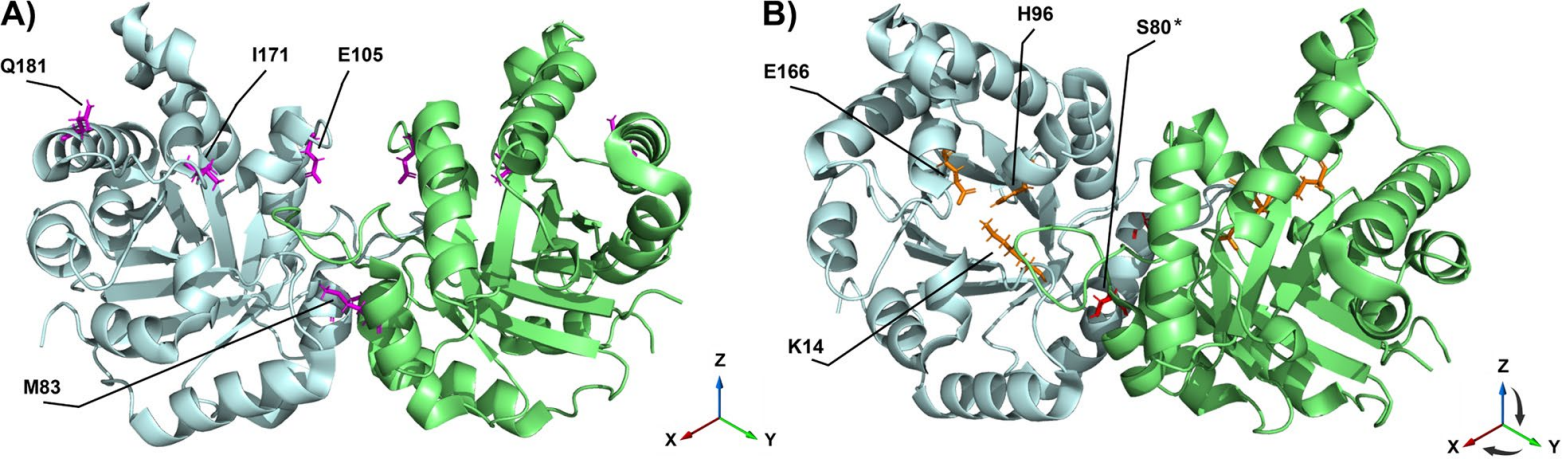
Structure of the human TPI protein homodimer with selected residues displayed. **A** Several key amino acids (magenta) are labeled that cause or model disease. The TPI^E105^ substitution site, which is associated with nearly all cases of human TPI deficiency, is noted. Also noted are the TPI^Q181^ and TPI^I171^ mutation sites which only cause disease when paired with a *TPI1*^*E105D*^ allele. Finally, the TPI^M83^ mutation site in the human TPI protein, which corresponds to the *Drosophila* TPI^sgk^ mutation site, is shown. Important to note is the proximity of both the TPI^E105^ and TPI^M83^ residues to the dimer interface. **B** Active site residues and the TPI^S80^ phosphorylation site. Active site residues TPI^K14^, TPI^H96^, and TPI^E166^ are displayed in orange. The TPI^S80^ phosphorylation site, which affects the nuclear localization of TPI, is displayed in red. The TPI^S80^ phosphorylation site is labeled on the opposite subunit as the active site residues are labeled. Figure created with the PyMOL Molecular Graphics System, Version 2.5.2, Schrödinger, LLC, using Protein Data Bank structure 4POC

Triosephosphate isomerase (TPI) is a highly conserved essential enzyme from bacteria to humans with a well-established role in glycolysis (Reynolds et al. 1971). TPI has been incredibly well-studied structurally and information about the structure and function of the enzyme was reviewed several years ago (Olivares-Illana et al. 2017). Mutations in the human *TPI1* gene, including the “common” *TPI1*^*E105D*^ mutation, result in a severe and untreatable glycolytic enzymopathy known as TPI Deficiency (TPI Df). TPI Df results in pediatric-onset anemia and severe multisystemic dysfunction characterized by neuromuscular impairment, presumably owing to a loss of glycolytic function and the resulting over-reliance on aerobic respiration for energy production (Schneider et al. 1965). However, less established are the functions TPI has beyond glycolysis. Does TPI have nuclear and/or non-catalytic functions? Several intriguing publications suggest TPI may have several functions independent of glycolysis and possibly independent of isomerase activity (Daniel et al. 2021; Jin et al. 2022; Liu et al. 2022; Rodriguez-Bolanos and Perez-Montfort 2019; Roland et al. 2015, 2013; Zhang et al. 2021). An overview of the structure of TPI with pertinent residues labeled can be found in Fig. 1.

## History of moonlighting

**Fig. 2:**
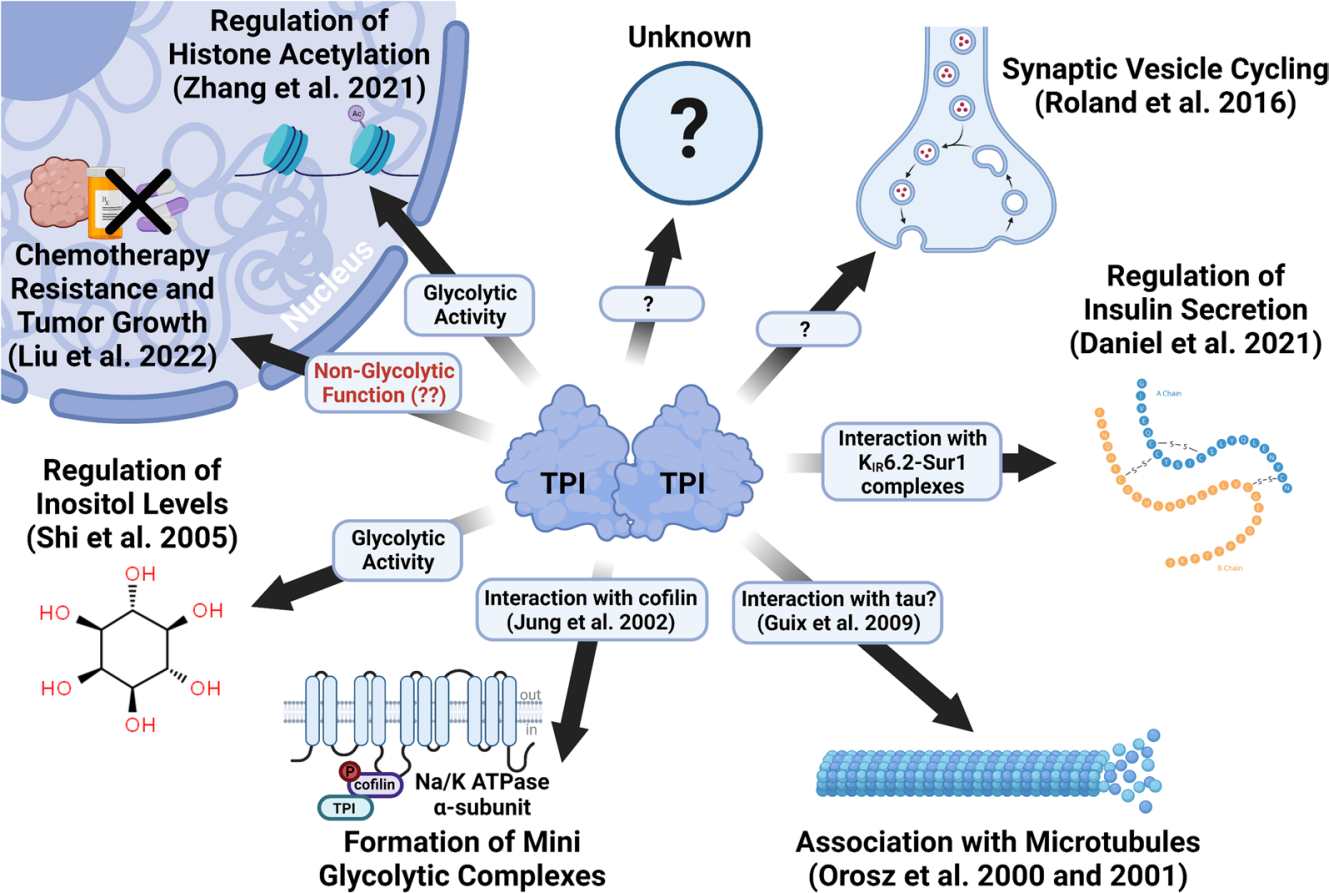
Published functions of triosephosphate isomerase (TPI) that possibly represent moonlighting. Specific functional effects of TPI are described at each node, as well as the proposed mechanism underlying this function, if known. Designated in red text is the conclusion drawn by Liu et al. (2022), which has not yet been supported experimentally. Figure created using BioRender.com

Genes encoding multiple protein isoforms is commonplace, especially in higher animals, due to the use of multiple promoters or alternative polyadenylation sites, RNA editing, alternative splicing and a host of post-translational modifications. Although these isoforms may encode a range of functions, these are not generally considered examples of gene sharing or what has been termed moonlighting functions (Freedman 1978; Gancedo et al. 2016). Gene sharing involves completely unrelated “moonlight” functions, the prototypical example being crystallins that both form the structural yet transparent lens of the vertebrate eye and a soluble protein capable of enzymatic and non-enzymatic functions (Piatigorsky et al. 1988; Piatigorsky and Wistow 1989). In the case of TPI, numerous functions have been discovered that have been reviewed previously (Rodriguez-Bolanos and Perez-Montfort 2019) but recent studies suggest the exent of *TPI1* gene sharing may still be coming into focus (Fig. 2).

## Evidence for a potential non-catalytic TPI function

A decade ago, the possibility that TPI has non-catalytic moonlighting roles was first theorized as an explanation as to why *Trichomonas vaginalis* has two fully functional *tpi* genes (Figueroa-Angulo et al. 2012). The phenomenon of potential TPI moonlighting roles was first reported experimentally in studies of TPI Df (Roland et al. 2013). The *TPI1*^*K14M*^ mutation encoding catalytically inactive TPI was able to genetically complement TPI Df phenotypes in a *Drosophila* model of the disease (*TPI*^*sgk*^*)* (Roland et al. 2013). These data support the possibility that TPI has important non-catalytic functions. Alternatively, the genetic complementation could also be the result of TPI^K14M^ stabilizing TPI^sgk^ heterodimers. The *TPI*^*sgk*^ mutation results in a protein with a small defect to catalytic activity and a large defect in protein stability. Thus, because TPI is known to function as a dimer, the basis of the observed complementation could be that the catalytically inactive TPI is stabilizing TPI^sgk^ in TPI^K14M^ -TPI^sgk^ heterodimers. Simply put, the non-catalytic function might be to stabilize its binding partner that has activity. However, TPI^sgk^ levels were not elevated in *TPI1*^*sgk/K14M*^ animals leaving the intriguing possibility that TPI^K14M^ was providing a non-catalytic TPI function that improved animal phenotypes (Roland et al. 2013). Interestingly, another TPI Df causing mutation, *TPI1*^*Q181P*^, was discovered in compound heterozygous TPI Df patients alongside *TPI1*^*E105D*^ (*TPI1*^*E105D/Q181P*^). The *TPI1*^*Q181P*^ mutation results in a protein with strongly reduced catalytic activity but increased thermal stability (VanDemark et al. 2022). When the *TPI1*^*Q181P*^ allele was paired with the *TPI1*^*E105D*^ allele, it resulted in an atypical and *less* severe form of the disease, the proposed basis of which is genetic complementation between these alleles (VanDemark et al. 2022). Understanding the basis of the genetic complementation observed in TPI Df is important to elucidate, especially considering newly discovered moonlighting functions and binding partners.

There are several lines of evidence that deficits in TPI catalytic activity alone are insufficient to cause disease. Recently, Segal et al. studied a murine model with the *TPI1*^*I171V*^ mutation (known previously as *TPI1*^*I170V*^) (Segal et al. 2019). This mutation strongly reduces catalytic activity, but mutant mice did not recapitulate neuromuscular symptoms of TPI Df, even though the mice did exhibit hemolytic anemia (Cabrera et al. 2018; Segal et al. 2019). These results demonstrate that loss of catalytic activity is sufficient to cause pathogenesis in erythrocytes that are reliant upon glycolysis for energy, however, loss of TPI catalytic activity is not the principal driver of the neuromuscular pathogenesis observed in TPI Df. Consistent with these findings it is known that TPI Df causing mutations, such as *TPI1*^*E105D*^, affect the stability of TPI resulting in severe phenotypes (Schneider et al. 1965; Vives-Corrons et al. 1978). When modeling the *TPI1*^*E105D*^ mutation within the mouse, the phenotype was found to strongly resemble the human disease symptomology and this phenotype was accompanied by a strong decrease in the amount of TPI protein (Myers et al. 2022). These data support the conclusion that it is loss of stability and lowered TPI levels (*affecting all TPI functions*) that underlie the disease and open up the possibility that loss of non-glycolytic functions of TPI may contribute to the disease, especially within the neuromuscular system. Indeed, several moonlighting roles have been identified, but the catalytic dependence of these roles is unknown (Daniel et al. 2021; Jin et al. 2022; Liu et al. 2022; Rodriguez-Bolanos and Perez-Montfort 2019; Zhang et al. 2021).

## Nuclear roles of TPI

Recently a publication by Liu et al. (2022) reports that TPI has nuclear functions with a critical role in cancer pathogenesis (Liu et al. 2022). This paper effectively demonstrates the importance of nuclear TPI localization in lung adenocarcinoma where TPI promotes tumor cell growth and migration. The authors demonstrate that TPI nuclear localization can be induced by oxidative stress alone, as well as by chemotherapeutic administration, which are novel and important findings. Additionally, it was reported that TPI nuclear localization enhances resistance to chemotherapeutics, which has substantial implications in the design of cancer therapies. The importance of these findings potentially goes beyond cancer biology. Another key conclusion worthy of highlighting is that mutant TPI^E105D^ protein (referred to in Liu et al. 2022 and other manuscripts as TPI^E104D^) seems to be capable of translocating into the nucleus. They demonstrated that TPI^E105D^ protein is capable of upregulating growth and migration to the same level as wild-type TPI and that nuclear localization is necessary for this effect (Liu et al. 2022). This suggests that the pathogenesis of TPI Df, or at least the most common presentation resulting from homozygous *TPI1*^*E105D*^, is not the result of a loss of nuclear translocation of TPI, which is important to know.

What is TPI doing in the nucleus? Is it functioning in glycolysis? As an isomerase? Liu et al. (2022) investigated whether the role of TPI in the nucleus is independent of its catalytic activity, which is certainly an important question. They concluded that nuclear TPI function was independent of activity; however, this claim cannot be substantiated with the experiments presented in this manuscript. A significant flaw in this otherwise important publication is the claim that the *TPI1*^*E105D*^ mutation results in a catalytically inactive TPI protein. In fact, where activity has been studied, the TPI^E105D^ protein has very little, if any, reduction in catalytic activity as reported in several studies (Orosz et al. 2009; Rodriguez-Almazan et al. 2008), including the manuscript cited by the authors (Cabrera et al. 2018). Instead, the TPI^E105D^ protein has a primary defect in stability and protein levels are reported to be decreased in TPI Df patient cells carrying a homozygous *TPI1*^*E105D*^ mutation (Cabrera et al. 2018; Hrizo et al. 2021; Orosz et al. 2009; Rodriguez-Almazan et al. 2008). Therefore, the catalytic dependence of the role of nuclear TPI cannot be assessed using the TPI^E105D^ protein. To properly address this question, a *TPI1* mutation that results in a protein lacking catalytic activity and exhibiting ~ normal stability should be used. Fortunately, just such a mutation exists thanks to the pioneering work of Drs. Komives, Knowles and colleagues (Lodi et al. 1994). The *TPI1*^*K14M*^ mutation (referred to as *TPI1*^*K11M*^, *TPI1*^*K12M*^, *TPI1*^*K13M*^ or *TPI1*^*Δcat*^ in different species and previous publications) would be an ideal mutation for these studies and would provide meaningful information about the necessity of catalytic function for the role of TPI in the nucleus (Lodi et al. 1994; Roland et al. 2013, 2015).

Interestingly, another recent paper highlighted a role for nuclear TPI in regulating histone acetylation that seems to be entirely dependent on its catalytic function (Zhang et al. 2021). In this paper, nuclear TPI was reported to regulate histone acetylation through modifying levels of TPI’s substrate, DHAP (Zhang et al. 2021). Specifically, nuclear TPI was associated with lower concentrations of DHAP, resulting in higher concentrations of acetate and higher levels of histone acetylation leading to significant transcriptional alterations (Zhang et al. 2021). While the regulation of histone acetylation appeared to be dependent on TPI catalytic activity, this paper also investigated interaction partners for TPI using tandem affinity purification and identified several novel interaction partners (Zhang et al. 2021). It has yet to be established what functional effects these TPI interactions serve and whether these functions rely on catalytic activity. Interestingly, one TPI interaction partner identified is peroxiredoxin 6 (PRDX6), which has been associated with cisplatin resistance and is interesting given the findings of Liu et al. (2022), Xu et al. (2019), Zhang et al. (2021). Also relevant, TPI was found to interact with the Y-box binding protein 1 (YBX1) transcription factor which regulates several growth-related genes (Murugesan et al. 2018; Zhang et al. 2021). Informative follow-up studies should evaluate the functional consequences of these TPI interactions and determine whether these functions are dependent on the catalytic activity of TPI.

## TPI signaling partners

The signaling pathways regulating and regulated by TPI have been coming into focus recently as well. Several lines of evidence suggest a link between TPI and mammalian target of rapamycin complex 1 (mTORC1) signaling. In Zhang et al. (2021), TPI was found to be regulated through an axis involving mTORC1 and cyclin-dependent kinase 2 (CDK2), where TPI phosphorylation by CDK2 at serine 80 (referred to as Ser117 in the paper referencing a predicted long TPI isoform) resulted in the translocation of TPI to the nucleus. Interestingly, another recent publication further highlighted the importance of mTORC1 in relation to TPI function, where it was shown that TPI promotes tumor growth and migration through an association with cell division cycle associated 5 (CDCA5) which led to the activation of phosphatidylinositol-3-kinase (PI3K) and subsequent activation of the protein kinase B (Akt/PKB) and mTORC1 pathways (Jin et al. 2022). These two manuscripts suggest that TPI is both regulated by the mTORC1 pathway and that TPI can itself regulate the mTORC1 pathway, at least in cancer. Another recent paper suggests that TPI’s substrate, DHAP, may be involved in upstream mTORC1 signaling (Orozco et al. 2020). The mechanisms and directionality governing the interaction between TPI and mTORC1 must be better investigated to understand this pathway, and studies should extend outside of the context of cancer so that the role of these interactions can be assessed in normal physiology. Aside from mTORC1 signaling, it was also recently reported that TPI protein is regulated by p62 (Jin et al. 2022). This is a novel and important finding when taken in the context of TPI deficiency, considering that p62 is known to play a role in protein degradation (Chen et al. 2020). Therefore, p62 could potentially represent a therapeutic target for stabilizing mutant TPI^E105D^ protein in TPI deficiency.

## Other moonlighting roles for TPI

Additional moonlighting roles for TPI have been identified. In 2021, it was discovered that TPI interacts with sulfonylurea receptor 1 and inward rectifying potassium channel 6.2 (Sur1-K_IR_6.2) complexes in the pancreas (Daniel et al. 2021). As a consequence of the interaction between TPI and Sur1-K_IR_6.2 complexes, insulin secretion was inhibited (Daniel et al. 2021). Several years ago, it was shown that TPI may be playing a role in the regulation of synaptic vesicle cycling (Roland et al. 2015). The catalytic dependence of both of these roles is unknown. DHAP, a substrate of TPI, has been shown to regulate inositol levels (Shi et al. 2005). Additionally, TPI has been shown to interact with microtubule-associated protein tau (tau) (Guix et al. 2009). Tau is known to function in stabilizing microtubules, and Guix and colleagues discovered that TPI and tau interact in Alzheimer’s disease. In this study, TPI and tau were confirmed to interact through co-immunoprecipitation experiments from cellular models as well as human brain tissue from Alzheimer’s disease patients (Guix et al. 2009). This could help explain the association of TPI with microtubules seen in some TPI Df patients (Orosz et al. 2000, 2001). Lastly, TPI has been shown to interact with cofilin at the sodium potassium ATPase to form a mini-glycolytic complex (Jung et al. 2002). Cofilin is also known to regulate the actin cytoskeleton, which raises the possibility that TPI may be interacting with cofilin in other contexts aside from the formation of mini-glycolytic complexes. The functional consequences of the interaction of TPI with these two regulators of cellular structure are not yet understood. Importantly, as TPI protein is highly and ubiquitously expressed it is identified in many co-immunoprecipitation experiments, the functional significance of which often requires independent validation.

Further moonlighting functions of TPI have been previously reviewed (Rodriguez-Bolanos and Perez-Monfort 2019). This review highlighted several key moonlighting functions and specifically focused on their clinical/veterinary importance. Specifically discussed was the in-depth relationship between TPI and cancer, the role of TPI as a virulence factor for pathogens as well as a potential allergen, a role for TPI in sperm function, and the complex relationship between TPI and Alzheimer’s Disease. We have not duplicated a discussion of these roles but have included them in a summary figure of proposed moonlighting functions (Fig. 2).

## Conclusions

As a ubiquitously expressed highly-abundant enzyme, the implications of TPI non-catalytic functions could be far-reaching and extend well beyond cancer and TPI Df. Determining the importance of all the functions of TPI and which ones require isomerase activity or possibly are structural in nature would advance the field significantly. This is a challenge for TPI that likely also extends to other highly conserved enzymes that may have adopted additional functions within our cells (Huberts and van der Klei 2010). Although TPI is well known as an example of a perfect enzyme whose catalytic rate is only limited by the rate of substrate diffusion (Knowles and Albery 1977), perhaps it is also a “perfect” example of a moonlighting protein that has numerous distinct cellular functions.

## Data Availability

Not applicable.

## Abbreviations

TPI: Triosephosphate isomerase
TPI Df: Triosephosphate isomerase deficiency
DHAP: Dihydroxyacetone phosphate
G3P: Glyceraldehyde-3-phosphate
PRDX6: Peroxiredoxin 6
YBX1: Y-box binding protein 1
mTORC1: Mammalian target of rapamycin complex
CDK2: Cyclin-dependent kinase 2
CDCA5: Cell division cycle associated 5
PI3K: Phosphatidylinositol-3-kinase
Akt & PKB: Protein kinase B
SUR1: Sulfonylurea receptor 1
K_IR_6.2: Inward rectifying potassium channel 6.2
